# Modeling workflows: Identifying the most predictive features in healthcare operational processes

**DOI:** 10.1371/journal.pone.0233810

**Published:** 2020-06-11

**Authors:** Colm Crowley, Steven Guitron, Joseph Son, Oleg S. Pianykh

**Affiliations:** 1 Massachusetts General Hospital, Boston, MA, United States of America; 2 Department of Statistics, University College Cork, Cork, Ireland; 3 Department of Statistics, Stanford University, Stanford, CA, United States of America; 4 Harvard Medical School, Massachusetts General Hospital, Boston, MA, United States of America; Universitat Politecnica de Catalunya, SPAIN

## Abstract

Limited resources and increased patient flow highlight the importance of optimizing healthcare operational systems to improve patient care. Accurate prediction of exam volumes, workflow surges and, most notably, patient delay and wait times are known to have significant impact on quality of care and patient satisfaction. The main objective of this work was to investigate the choice of different operational features to achieve (1) more accurate and concise process models and (2) more effective interventions. To exclude process modelling bias, data from four different workflows was considered, including a mix of walk-in, scheduled, and hybrid facilities. A total of 84 features were computed, based on previous literature and our independent work, all derivable from a typical Hospital Information System. The features were categorized by five subgroups: congestion, customer, resource, task and time features. Two models were used in the feature selection process: linear regression and random forest. Independent of workflow and the model used for selection, it was determined that congestion feature sets lead to models most predictive for operational processes, with a smaller number of predictors.

## Introduction

The quality of patient care is largely determined by our ability to manage its resources [1]. As patient footfall in hospitals continues to rise [2–4], hospitals find themselves under perpetual pressure to maintain and improve patient care standards. A traditional approach to this would be to keep offering more services but, as with many other industries, it is not feasible for healthcare providers to continually add more work to already limited resources.

As a result, it becomes imperative to optimize and redistribute the operational systems already in place, while avoiding negative impacts on quality of care, safety, and patient satisfaction [5]. A large part of this work can be done by predicting critical operational events–such as patient wait times, exam volumes, and workload surges–to improve on-time resource management and performance [6–8]. In particular, predicting workload surges and wait times in advance can be used to redistribute limited workforce more efficiently. For instance, previous research highlights the importance of delay time predictions in emergency departments (ED) [9,10]; with statistical evidence suggesting that keeping patients updated on their status made the ED stay more bearable and subsequently deterred patients from leaving before receiving treatment. Furthermore, wait time prediction was found to beneficially influence customer satisfaction in other industries, according to [11–13] and [14].

However, all these workflows and processes are commonly influenced by multiple diverse factors, which makes them too complex to be tracked by humans. Therefore, advanced predictive models are required to provide reliable results, and variables must capture the local environment to keep the models accurate. As a result, numerous feature sets and modelling approaches have been proposed in previous literature for the most common workflow prediction types, such as wait/delay time predictions. These included the average wait times for the last *k* customers [15], queue-length-based and delay-history based features with time varying components [16], model-based features [17,18] and historic wait times [19]. The authors of these approaches used a range of workflow modeling paradigms, from time series to queueing theory, each with its own advantages and limitations. This variety of possible approaches leads to the practical challenge of choosing the best, and studying whether several of them can be united into a single stronger predictive mode.

Nowadays, machine learning offers a new powerful opportunity to model operational events with the ability to capture complex data and environment variabilities. Moreover, using the unifying machine learning paradigm, we can view all previously proposed modeling variables as different choices of predictive feature sets. It is common knowledge that the quality of machine learning models is dependent on the choice of their features [20]. Then, can we identify a common set or type of features for predicting healthcare operational processes?

The main goal of our work was to answer this question.

## Methods

### Data collection and compilation

The importance of accurately predicting wait and process delay times can hardly be overstated, with benefits ranging from improved customer satisfaction to optimal resource allocation. Therefore, we have chosen this task to study the best predictive feature sets. Our work took place in a large academic center with close to one million patient examinations per year. To study different operational scenarios, we considered the two most common prediction tasks: predicting wait time for walk-in patients and predicting process delay time for scheduled patients.

To identify the best predictive features for wait/delay time with the least bias towards any particular site, we considered four differently organized outpatient imaging facilities: F1 (magnetic resonance imaging), F2 (ultrasound imaging), F3 (computed tomography), and F4 (X-Ray). F1 and F2 had scheduled appointments, and as a result servicing of the queue is not necessarily FIFO. F3 was mostly scheduled, with a few walk-ins interspersed. F4 was walk-in patients only.

Real-time and historical wait/delay times were taken from our Hospital Information System (HIS). For F1, F2 and F3 we predicted delay times as patients were arriving based on a schedule. Here, delay time was defined as the difference between scheduled time of the exam and the time the exam began (for the patients seen before their scheduled time, their delay time was a negative number.) In the case of F4, we predicted wait times, which is the difference between the arrival time of the patient and the time the exam began (always positive by definition). Although there are intrinsic differences between the scheduled and walk-in workflows, they share two fundamental properties: (1) The same patient experience, when the patient perceives any wait (whether it be from a scheduled exam or walk in exam) as their wait time; (2) The same staff experience, when any idling and delays should be reduced to ensure steady patient processing.

Facility data used in our analysis included all patient visit records from November 2017 to July 2019. All patient-identifying data was completely excluded from the dataset and subsequent analysis, and all features were aggregated across multiple patients per each visit time, to remove any patient-specific information. There were, on average, between 25 (F1) and 120 (F4) visits per day at the facilities which amounted to 15494 observations for F1; 36154 for F2; 30173 for F3; 69469 for F4.

### Feature set

It’s important to realize that the role of any single feature in a model does not depend on this feature alone, but also on the other features included in the model. As a result, looking for the best process predictors means identifying the best predictor sets, rather than picking best features individually.

Thus, by testing several models with different combinations of features, we aimed at discovering the best *feature sets* as the combination of features that make a good model. Looking for best sets instead of single features certainly complicates the task, as the number of all possible subsets grows exponentially as the number of features increases. Moreover, there may be many feature subsets with very similar predictive quality. However, if some feature sets can be identified as consistently associated with superior model quality, it can greatly simplify optimal model design, and provide more insights into the key process drivers.

With this rationale, our main goal was to consider all previously suggested features that we have found in the earlier research, as well as the features discovered by our group, to see if any particular feature subsets can be identified as the most predictive for operational events. 84 features were collected for analysis, which were categorized by five feature groups: congestion, customer-specific, resource-specific, task-specific and time features, as illustrated in Table 1.

**Table 1:** Feature set divided into 5 groups.

Group	Name	Description
Congestion	LineCount0Strict	Number of patients in line with scheduled times after current time.
	LineCount0/1/2/3/4	Number of patients in line measured when a patient arrives, 15, 30, 45 & 60 minutes before.
	FlowCount30/60	Number of patients starting exams in the 30- and 60-minute window before patient arrived.
	ScheduledFlowCount30/60	Number of patients scheduled in the 30- and 60-minute window before patient arrived.
	FutureFlowCount30/60	Number of patients scheduled in the 30- and 60-minute window after patient arrived.
	AheadCount	Number of patients scheduled before current patient for the day.
	IsFirst	First scheduled patient for the day.
	IsLast	Last scheduled patient for the day.
	NoneInLine	No patients in line.
	SumWaits	Sum of the wait times for patients in line.
	NumCustomersLast30/60/120	Number of customers who have arrived in the last 30, 60 & 120 minutes.
	NumScheduledNextSlot	Number of patients scheduled in next slot.
	NumScheduledNext60	Number of people scheduled in next 60 minutes.
	AvgWaitForDay	Average delay/wait for patients for that day.
	NumCompletedInLast30/60/120	Number of exams completed in last 30, 60 and 120 minutes.
	NumCompletedToday	Number of exams completed up to current of day.
	DelayedInLine	The number of patients in line who are delayed.
	MinTime	Minimum wait time for the day.
	MaxTime	Maximum wait time for the day.
	DelayCount	Number of delayed exams up to current time of day.
	DelayCountLastHour	Number of delayed exams in last hour.
	AvgWaitLast30/60/120	Average wait time last 30, 60 and 120 minutes.
	SumTimeToCompleteNextSlot	Expected time to completion of exams in next slot.
	SumTimeToCompleteNext60	Expected time to completion of exams scheduled in next hour.
	InProgressSize	Number of exams in progress for facility.
	SumTimeToCompleteInProgress	The sum of the expected times to complete of the exams in progress
	NoneCompleted	No exams completed that day.
	NoneInProgress	No exams in progress.
	SumInProgress	Sum of length of time exams have been in progress.
	MostRecent1/2/3/4/5	Delay/wait time for most recent patient, 2^nd^, 3^rd^, 4^th^ & 5^th^ most recent patients.
	AvgWaitLast2/4/8Customers	Average wait for the last 2, 4 and 8 customers.
	Median5	Median delay/wait time for 5 most recent customers.
	NumAddOnsToday	Number of people who have been added to the schedule for today.
	NumAddOnsLast60	Number of people who have been added to the schedule in last 60 minutes.
	SumHowEarlyWaiting	Sum of how early the patients in line are for their appointment.
	AvgHowEarlyWaiting	Average of how early the patients in line are for their appointment.
	SumDelayWaitingInLine	Sum of delays/waits of patients in line.
	SumDelayInProgress	Sum of delays/waits of exams in progress.
Customer	AvgAgePeopleWaiting	Average age of the patients in line.
	OutpatientWaitingCount	Number of outpatients waiting in line.
Resource	NumScannersInUseToday	Number of scanners in facility that have been used on that day.
Task	WithContrastCountWaiting	Number of patients waiting for an exam with contrast.
	WithandWithoutContrastCountWaiting	Number of patients waiting for an exam with and without contrast.
	WithContrastCountInProgress	Number of exams in progress with contrast.
	WithandWithoutContrastCountInProgress	Number of exams in progress with and without contrast.
	ExpectedDelayNextExam	Expected delay of the next scheduled exam.
	SumDelayWaitingByExamCode	Sum of delays of patients in line by exam type.
	AvgWaitByTaskTypeLine	Average waits of patients in line by exam type.
	SumWaitByTaskTypeLine	Sum of waits of patients in line by exam type.
	MSKCount	Number of patients waiting for musculoskeletal exam.
	CardiacCount	Number of patients waiting for cardiac exam.
	VascularCount	Number of patients waiting for vascular exam.
	AbdominalCount	Number of patients waiting for abdominal exam.
	NeuroCount	Number of patients waiting for neuro exam.
	PediatricCount	Number of patients waiting for pediatric exam.
	ThoracicCount	Number of patients waiting for thoracic exam.
Time	DayOfYear	The day of the year the exam is scheduled.
	Month	The month of the year the exam is scheduled.
	DayOfWeek	The day of the week the exam is scheduled.
	StartTime/2/3/4	Hour of arrival, and 2^nd^, 3^rd^ & 4^th^ powers of hour of arrival to account for nonlinear trends.
	BeforeSlot	Time since last appointment slot.
	AfterSlot	Time until next appointment slot.

Note that some features were not directly transferrable to all types of workflows we had: for example, features containing information about scheduled appointments were not applicable to walk-in F4, and some examination-specific features available in F1 and F3 (such as CardiacCount and NeuroCount) were not applicable to F2. In such cases, some feature values could be extended by using defaults or proxies (such as using arrival time in F4 as a substitute for the scheduled time), to create a universal, scalable, facility-independent feature set.

Also note that we did not use individual patient predictors (such as patient age or time to complete for each patient). Patients can arrive in different orders and exhibit different behavior, and we intentionally wanted our models to be independent of individual patient properties or preferences. Instead, we are predicting expected delay/wait for the next patient, whoever it will be. We have, however, included similar features for patients currently being serviced by the system (such as SumTimeToCompleteInProgress).

The counts for individual exams (CardiacCount, NeuroCount, etc.) are based on the patients waiting in line for a single type of exam. However, some patients come for multiple exams which are performed directly after one another. For instance, some patients come to F3 and have a chest, abdominal and pelvis scan consecutively. This combination of exams will have a unique exam code and therefore these patients will not be included in the individual exam features. It is most common for patients to come for a single type of exam and therefore we have only included counts for the individual exams.

## Models

To identify the most predictive features, independent of possible model bias, two entirely different models were considered for each facility: linear regression and random forest. Linear regression was selected as the most classical model type, while random forest was used because of its ability to model highly nonlinear dependencies, variable interactions, and tree-like decision making, so common in operational logic. The dependent variable was delay time for F1, F2 and F3; and wait time for F4. In both cases, the dependent variable was continuous by nature and denominated in minutes.

All analysis was carried out using R-3.6.1 (www.r-project.org).

### Stepwise feature selection with linear regression

**Fig 1:**
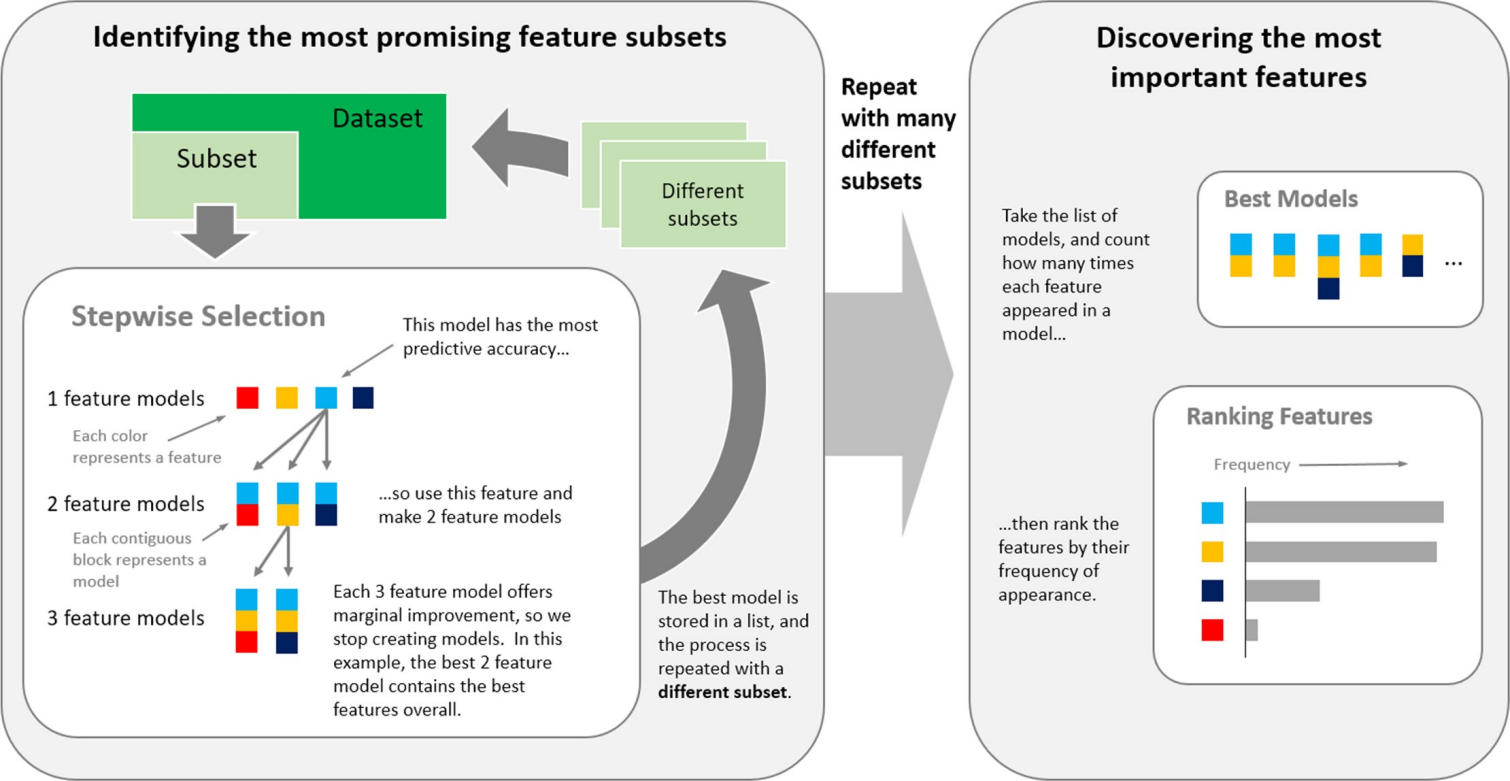
Feature selection process for linear regression, which was performed separately for each facility, on multiple data subsets. Features were added to the best model, at each step, based on predictive performance. This process was repeated 100 times, each with different subsets of the data, to avoid data bias.

To avoid exponentially expensive best subset search, stepwise linear regression was used to pre-identify the most promising feature subsets, for each of the four facilities F1-F4 (Fig 1). However, to avoid data bias in a single “greedy” stepwise selection result, the selection process was repeated on multiple subsets of the original data (Fig 1). For each facility, a random consecutive sample of six months of visits was used as the training (sub)set; and a further consecutive two weeks of visits were used as the test set. At each step, the feature that offered the largest decrease in out-of-bag mean-squared error (MSE) was added to the linear regression model based on predictions made using 10-fold cross validation on the training set.

**Fig 2:**
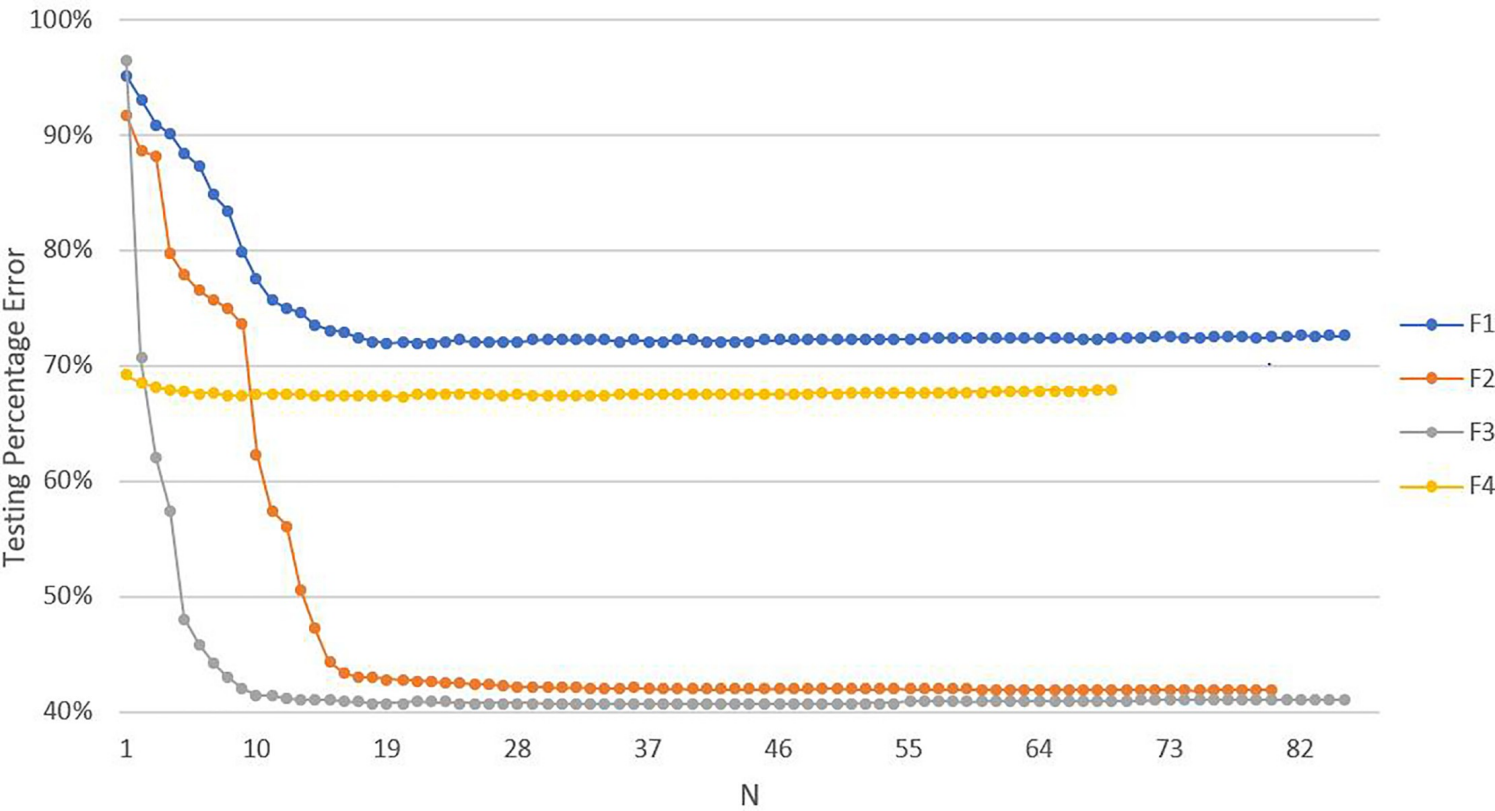
Reduction in the percentage of test error (Testing Percentage Error) as features (N) are added to the linear regression model. Testing error refers to the ratio of the test MSE of the model of size N to the test MSE of the model with the intercept only; with a Testing Percentage Error of 100% indicating the error made by the intercept only model. As features are added to the model, we can see that after 20 features the test error for all four facilities has plateaued, with F1 being the last facility to do so.

Fig 2 shows the percentage reduction in model test error (Testing Percentage Error) as more model features (N) are added to the linear regression model for each facility. Test error was the resulting MSE after making predictions, using the model of size N, on the test set. The percentage reduction in test error was calculated by comparing predictions made by the linear regression model of size N to predictions made by the linear regression model containing only the intercept (average delay/wait time in this case). A Testing Error value of 100% indicates the error made by the intercept only model. As is clear from Fig 2, the test error improves at a different rate for each facility, with F1 requiring the most features (20) for the test error to plateau. Therefore, 20 features were added to each model, for each facility, in order to make the feature sets directly comparable.

This process was repeated for 100 train/test data pairs, for each facility, and the number of times each variable appeared in the 100 linear regression models was recorded. In this way, we significantly broadened the plain stepwise search, identifying many more potential good feature set candidates to consider.

### Random forest-based feature selection

The second model considered was random forest. Before fitting each random forest, hyperparameters for the number of variables to be considered at each split, maximum depth of the trees and number of trees per random forest were optimized using 10-fold cross validation. Since stepwise feature selection cannot be done with random forests (for example, it is hard to build forests with only one or two variables), we took another feature set pre-selection approach. First, a random forest was fitted for each facility. Then the built-in MSE-driven importance feature [21] in the random forest algorithm was used to record the effectiveness of each feature. This was repeated for 100 train/test pairs, for each facility, and the features were ranked based on cumulative importance.

## Results

### Most predictive features for each facility

**Fig 3:**
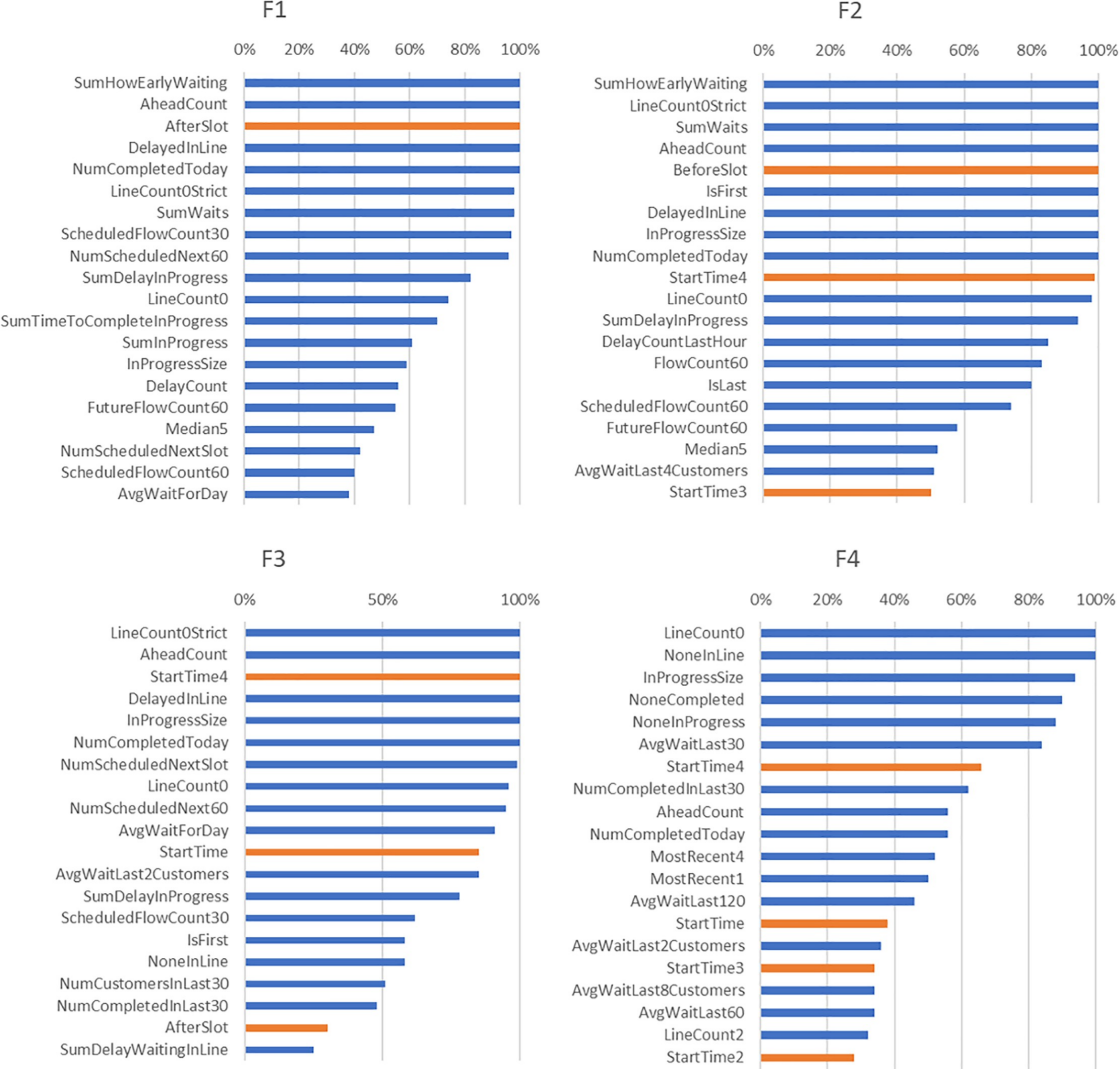
Features selected in the linear regression model for each facility. The congestion features are highlighted in blue and the time features highlighted in orange; no other feature types were identified by the selection algorithm. 100% on the horizontal axis indicates that a variable was selected in all the linear regression models for that facility.

Fig 3 provides a summary of features identified as the most frequently associated with the optimal linear regression models. Note that the majority of these features belong to the congestion feature group (colored in blue), with a few from the time feature group (colored in orange).

Given that most of the features are from the congestion group, we sought about further reducing the common size of the optimal feature set without a deterioration of more than two minutes in predictive quality for each facility. The comparison of predictions made by the linear regression model containing the full feature set, to the predictions made by the linear regression model containing the reduced, best 10-feature set, are shown in Table 2.

**Table 2:** Predictive performance, measured by mean absolute error, of reduced feature and full linear regression models for each facility. The mean absolute errors of both models are denominated in minutes.

Facility	Full feature set (minutes)	Best 10-feature set (minutes)
F1	17.60	19.20
F2	8.55	9.32
F3	22.95	23.07
F4	3.91	3.91

As displayed in Table 2, the difference in predictive quality between the model with the full feature set and the model with the best 10-feature set was under two minutes for F1, and even less for F2-F4. Therefore, we decided to study only the best 10-feature sets chosen for each facility. Unsurprisingly, the difference in the predictions made by the two sets was greatest for F1 (1.6 minutes), as it required the most features for the test error to plateau (Fig 2). Additionally, the similarity in the predictions made by the models containing the two different feature sets justifies identifying the best predictive features based on frequency of appearance.

We also noticed that there was significant overlap between the features selected for F1, F2, and F3 (Fig 3), most likely because the three facilities are primarily schedule driven. Therefore, we considered the union of the top 10 best predictive features from each of the three facilities, resulting in 17 features (Table 3).

**Table 3:** Union of features for F1, F2 and F3 as selected by forward stepwise linear regression.

Best features for F1, F2 and F3	
Feature	Group
LineCount0Strict	Congestion
AheadCount	Congestion
StartTime4	Time
DelayedInLine	Congestion
InProgressSize	Congestion
NumCompletedToday	Congestion
NumScheduledNextSlot	Congestion
LineCount0	Congestion
NumScheduledNext60	Congestion
AvgWaitForDay	Congestion
SumHowEarlyWaiting	Congestion
AfterSlot	Time
SumWaits	Congestion
ScheduledFlowCount30	Congestion
SumDelayInProgress	Congestion
BeforeSlot	Time
IsFirst	Congestion

As mentioned previously, it was interesting to observe that almost all the best predictive features in this list turned out to be congestion-related and did not contain any exam-specific features. Only two different types of line count appeared, both of which are measured immediately on arrival of the patient, indicating that continued measurement of line size before the patient arrives for their exam is not necessary. Additionally, there were a few features selected that provide information about the next scheduled slot (NumScheduledNextSlot, NumScheduledNext60 and AfterSlot). Furthermore, delay- and wait-history-based features were prominent in the selection which justifies their inclusion as suggested in [16].

Walk-in F4 was considered separately to the union of features given in Table 3 as several variables selected for F1, F2 and F3 depended on information from a schedule which, as mentioned, was not part of the F4 workflow. Table 4 gives the best predictive features for F4 as selected by the linear regression model.

**Table 4:** Best predictive features for F4 as selected by linear regression.

Best Features for F4	
Feature	Group
LineCount0	Congestion
AheadCount	Congestion
NoneInLine	Congestion
NoneInProgress	Congestion
NoneCompleted	Congestion
StartTime4	Time
InProgressSize	Congestion
NumCompletedToday	Congestion
NumCompletedInLast30	Congestion
AvgWaitLast30	Congestion

Again, it became evident that the best F4 predictors remained congestion variables even though F4 was a completely different, walk-in workflow.

### Feature transferability

Transferability of features is one of the most practically important properties of machine learning applications: we would like model feature sets to remain optimal or nearly optimal in different environments, so that the time-consuming feature engineering does not need to be repeated at each site. Feature transferability also serves as an indicator that the selected feature set captures the essence of the underlying process.

Therefore, the transferability of best predictive features across facilities was investigated, to discover if features selected by the algorithm for one facility could be used to make predictions for the other facilities without large differences in predictive performance. The top 10 features from each facility were used to predict delay/wait times for the other facilities and the predictions from the transferred 10-feature set were then compared to predictions made using the facility optimal 10-feature set. We also used the union of features from Table 3 to make predictions for each facility. Table 5 gives the transferability of features that were selected by linear regression.

**Table 5:** Transferability of features selected by linear regression: How well the features selected as optimal for each facility (columns) approximate the data from each facility (rows). The ratio represents the mean of the predictions made by the transferred feature set compared to the predictions made by the facility optimal feature set. A ratio of 1 indicates that the predictions are equivalent and a ratio of 1.06 indicates that the predictions made by the transferred set are 6% worse than the predictions made by the facility optimal feature set.

		Features				
		F1	F2	F3	F4	*Union*
**Data**	F1	-	1.14	1.09	1.15	*1*.*02*
	F2	1.12	-	1	1.07	*1*
	F3	1.08	1.08	-	1.14	*1*
	F4*	1.02	1.02	1.01	-	*1*.*02*

*When using F1, F2 and F3 features to predict F4, the best 10 predictive features that were suitable for F4 were used. When using the Union to predict F4, there were 8 features that were applicable to F4.

As seen in Table 5, the features we identified for each facility proved to be transferable, but the optimal features from the Union set (Table 3) provided an exceptionally good match for all four facilities, including walk-in F4. Thus, although F4 was originally considered separately, the results in Table 5 demonstrate that the features in Table 3 are transferable across inherently different workflows.

The same experiment was repeated using random forest: the predictive performance of the random forest with the full feature set was compared to a random forest fitted with the best 10 predictive features for each facility, selected based on cumulative importance (Table 6). A reduced set of 10 features was chosen in this case in order to make it directly comparable to the feature sets selected by the linear regression model. With random forest, it is even more apparent than linear regression that, using a reduced model with only one eighth of the total feature set can produce a model nearly as accurate as the full model.

**Table 6:** Predictive performance, measured by mean absolute error, of reduced feature and full feature random forest models for each facility. The mean absolute errors of both models are denominated in minutes.

Facility	Full feature set (minutes)	Best 10-feature set (minutes)
F1	19.23	18.24
F2	9.92	10.02
F3	24.47	24.55
F4	3.98	3.98

The same unification process was then carried out for the variables that were identified by the random forest. The union of the top 10 features for F1, F2 and F3 are given in Table 7.

**Table 7:** Union of features for F1, F2 and F3 as selected by importance in random forest.

Best features for F1, F3, and F2	
Feature	Group
LineCount0Strict	Congestion
AheadCount	Congestion
StartTime	Time
StartTime2	Time
StartTime3	Time
StartTime4	Time
NumCompletedToday	Congestion
DelayedInLine	Congestion
AvgWaitLastK3Customers	Congestion
Median5	Congestion
SumDelayInProgress	Congestion
BeforeSlot	Time
SumWaits	Congestion
DelayCount	Congestion

As seen in Table 7, the nature of the features selected by the random forest was very similar to those selected by the linear regression. There were eight features in common between the two sets of features: LineCount0Strict, AheadCount, StartTime4, NumCompletedToday, DelayedInLine, SumWaits, SumDelayInProgress and BeforeSlot. Again, the congestion features had a notable presence with the absence of any task specific features. Interestingly, the random forest also selected the four StartTime features from the time group. Only line sizes when the patient arrived were deemed important, which was also the case with the linear regression; and the random forest placed almost all emphasis on delay- and wait-history-based features rather than taking future exams into account.

Again, the F4 features were considered separately to the union of features for random forest as there were features selected for F1, F2 and F3 that did not apply to F4. The features DelayedInLine and DelayCount were not suitable as wait time, and not delay time, was measured for F4. However, SumDelayInProgress was relevant as it was measuring the sum of the waiting times of the people currently in exams. Given that arrival time of patients was used as scheduled time for F4 patients, delay in this case was measuring the difference between arrival time and begin time of the exam, which is the length of time the patient waited before the exam began. Table 8 gives the top 10 best predictive features as selected by the random forest for F4.

**Table 8:** Best predictive features for F4 as selected by random forest.

Best Features for F4	
Feature	Group
LineCount0	Congestion
AheadCount	Congestion
StartTime	Time
StartTime2	Time
StartTime3	Time
StartTime4	Time
SumTimeToCompleteInProgress	Congestion
SumDelayInProgress	Congestion
NumCustomersInLast30	Congestion
SumWaits	Congestion

Like linear regression, F4 was predicted with features mainly from the congestion group even though it is completely different to F1, F2 and F3. The transferability of the features selected by the random forest was investigated and the results are presented in Table 9.

**Table 9:** Transferability of features selected by random forest. Given the random nature of the tree-based algorithm, 95% confidence intervals are provided in parentheses. The ratio represents the mean of the predictions made by the transferred feature set compared to the predictions made by the facility optimal feature set (a ratio of 1.06 indicates that predictions made with the transferred set are 6% worse).

		Features				
		F1	F2	F3	F4	Union
**Data**	F1	-	1 (0.9,1.17)	0.99 (0.91,1.08)	1.04 (0.95,1.15)	0.97 (0.88,1.07)
	F2	1.03 (0.95,1.1)	-	1 (0.97,1.03)	1.04 (0.96,1,13)	0.98 (0.9,1.02)
	F3	1.05 (0.99,1.13)	1.04 (0.98,1.11)	-	1.1 (0.99,1.21)	1 (0.96,1.07)
	F4*	1.02 (0.97,1.06)	1 (0.96, 1.05)	1.02 (0.99,1.07)	-	0.99 (0.96,1.05)

*When using F1, F2 and F3 features to predict F4, the best 10 predictive features that were suitable for F4 were used. When using the Union to predict F4, there were 12 features that were suitable for F4.

As seen in Table 9, the features are again transferable between F1, F2 and F3 and in some cases the Union set of features produces better predictions than the facility optimal features. A potential explanation for this is that the Union contains 14 features and is larger than each facility optimal 10-feature set allowing the random forest to split on more optimal variables. F4 features perform relatively well when predicting the other facilities despite the different system dynamics.

Additional to the investigations into the transferability of features between facilities, the transferability of features between the two models was then investigated. The features that were selected by the linear regression were used in the random forest and vice-versa. Using the union of 14 best predictive features selected by the random forest algorithm, in a linear regression model, resulted in a mean prediction error of 18.32 minutes for F1, 9.93 minutes for F2, 23.93 minutes for F3. Notably, the prediction error here for F1 is slightly better than the prediction error using the top 10 features as selected by the linear regression. This is to be expected as the prediction performance for F1 does not plateau until after 20 features. Using the union of 17 best predictive features selected by the linear regression model, in the random forest, resulted in mean prediction error of 18.08 minutes for F1, 9.92 minutes for F2, 23.92 minutes for F3. The results indicate that, although the two models selected different features, the features were transferable without losing predictive power as they were of the same nature.

To further investigate the generalizability of the feature set across healthcare operational processes, the best predictive features for another vital workload indicator–waiting line size–were selected using the cumulative importance method of the random forest. This resulted in the set of best predictive features, once again, being comprised of features from the congestion group. The selection of congestion features, not only independent of selection method and facility, but also independent of operational process, emphasizes their importance in healthcare workflow predictions.

## Conclusions

In this study, a large set of conceptually different features was gathered from the previous research and our independent work, to identify the most predictive feature sets for common healthcare operational processes. To exclude model and facility bias, two different machine learning models (linear regression and random forest) were applied to four different operational facilities (workflows). Optimal feature selection in both models demonstrated that the most predictive operational process feature sets are made up of mostly congestion features (with a few features from the time group). This finding was further emphasized by cross-model validation, when the features selected by one model were used in the other, still producing (nearly) optimal results, and transferability of congestion based operational models. The same importance of congestion predictors was confirmed by considering different types of processes and operational predictions. Even though F4, a walk-in facility, was an inherently different operational system compared to scheduled F1, F2 and F3; congestion features were identified as the most predictive for both scheduled and walk-in models.

As a result, it can be concluded that the best feature set for the most common operational models in healthcare can be built from a rather standard list of congestion features. This is non-trivial: for instance, the inclusion of environment and task specific features (such as examination types) in our case did not seem to improve the accuracy provided by the congestion feature set. This means that one can avoid complex model customization for operational workflows running inherently different tasks. Instead, a small set of very standard congestion features can be used to create accurate and scalable machine learning models, naturally generalizable to different operational environments.
